# Rehearsal-Free Continual Learning for Emerging Unsafe Behavior Recognition in Construction Industry

**DOI:** 10.3390/s25216525

**Published:** 2025-10-23

**Authors:** Tao Wang, Saisai Ye, Zimeng Zhai, Weigang Lu, Cunling Bian

**Affiliations:** 1 Department of Campus Security, Ocean University of China, Qingdao 266000, China; wt@ouc.edu.cn; 2 Department of Education, Ocean University of China, Qingdao 266000, China; yesaisai@stu.ouc.edu.cn (S.Y.); zhaizimeng@stu.ouc.edu.cn (Z.Z.); luweigang@ouc.edu.cn (W.L.)

**Keywords:** rehearsal-free continual learning, emerging unsafe behavior recognition, construction industry, prompt

## Abstract

In the realm of Industry 5.0, the incorporation of Artificial Intelligence (AI) in overseeing workers, machinery, and industrial systems is essential for fostering a human-centric, sustainable, and resilient industry. Despite technological advancements, the construction industry remains largely labor intensive, with site management and interventions predominantly reliant on manual judgments, leading to inefficiencies and various challenges. This research emphasizes identifying unsafe behaviors and risks within construction environments by employing AI. Given the continuous emergence of unsafe behaviors that requires certain caution, it is imperative to adapt to these novel categories while retaining the knowledge of existing ones. Although deep convolutional neural networks have shown excellent performance in behavior recognition, they traditionally function as predefined multi-way classifiers, which exhibit limited flexibility in accommodating emerging unsafe behavior classes. Addressing this issue, this study proposes a versatile and efficient recognition model capable of expanding the range of unsafe behaviors while maintaining the recognition of both new and existing categories. Adhering to the continual learning paradigm, this method integrates two types of complementary prompts into the pre-trained model: task-invariant prompts that encode knowledge shared across tasks, and task-specific prompts that adapt the model to individual tasks. These prompts are injected into specific layers of the frozen backbone to guide learning without requiring a rehearsal buffer, enabling effective recognition of both new and previously learned unsafe behaviors. Additionally, this paper introduces a benchmark dataset, Split-UBR, specifically constructed for continual unsafe behavior recognition on construction sites. To rigorously evaluate the proposed model, we conducted comparative experiments using average accuracy and forgetting as metrics, and benchmarked against state-of-the-art continual learning baselines. Results on the Split-UBR dataset demonstrate that our method achieves superior performance in terms of both accuracy and reduced forgetting across all tasks, highlighting its effectiveness in dynamic industrial environments.

## 1. Introduction

Construction industry, notorious for its high rate of safety-related accidents due to human behaviors, exemplifies its urgent need for safety protection. Construction projects typically involve complex interactions between personnel and machinery, each presenting unique and dynamic challenges. Ensuring safety within the construction industry has been a persistent global concern, marked by significantly higher injury and fatality rates compared to other sectors [1]. The importance of maintaining safety in construction has become even more pronounced with the sector’s recent revitalization and the heightened demand for infrastructure development. Despite comprehensive efforts to improve safety, the incidence of accidents and fatalities in construction has remained consistently high or plateaued in recent years [2]. Research indicates that a substantial proportion of construction accidents can be directly linked to unsafe behaviors by workers, such as neglecting to use fall protection equipment [3]. Despite initiatives aimed at modifying these behaviors through training, the effectiveness has been limited, with workplace negligence and oversight continuing to significantly contribute to the occurrence of incidents. Therefore, this paper proposes to develop an automated tool for preventing safety incidents based on recognizing unsafe behavior, which is crucial at the intersection of construction industry safety management and AI [4,5,6].

Recent years have witnessed increasing attention to automated safety monitoring in construction environments using computer vision and deep learning [7]. Early approaches primarily relied on handcrafted features or static pose analysis to detect unsafe acts, such as improper equipment use or fall hazards [8]. With the advent of large-scale visual datasets and pre-trained convolutional backbones, deep models have significantly improved recognition accuracy [1,9]. Nevertheless, these methods typically assume a fixed set of behavior categories and require retraining when new unsafe patterns emerge.

To overcome this limitation, continual learning has emerged as a promising direction that enables models to incrementally acquire new knowledge while retaining previously learned tasks [10]. Different types of existing approaches strive to preserve and extend acquired knowledge during the continual learning process. Regularization-based methods mitigate catastrophic forgetting by regularizing critical parameters for learned tasks, although their performance in more challenging class-incremental settings is often suboptimal [11]. Architecture-based methods allocate isolated parameters by expanding or segmenting the model to encode knowledge for each task, but these generally require a substantial increase in additional parameters [12]. Rehearsal-based methods store data from learned tasks in a rehearsal buffer to train alongside current tasks, proving highly effective even in class-incremental settings [13]. However, the efficacy of rehearsal-based methods diminishes with reduced buffer size and they are not suitable for scenarios where data privacy is a concern. Despite their success in general vision tasks, few studies have explored how CL can be applied to safety-critical domains such as construction, where data privacy and continual task evolution are both crucial. Moreover, unsafe behavior recognition is not only a visual classification problem but also involves dynamic and context-dependent cues [14]. Recent multimodal or context-aware frameworks [15,16] show that integrating scene context, pose, and temporal information can enhance risk understanding. However, these methods remain static and do not adapt to newly emerging unsafe behaviors over time. Therefore, there is a pressing need for a rehearsal-free continual learning framework tailored to the evolving nature of construction safety data, which motivates the present study.

Building on prior research, this paper proposes a flexible and efficient recognition method capable of expanding the scope of unsafe behaviors while maintaining the recognition of both new and old categories. To our knowledge, our approach is the first to tackle continual learning in the context of emerging unsafe behavior recognition within the construction industry. The proposed method incorporates complementary prompts into the pre-trained model, facilitating the acquisition of both task-invariant and task-specific knowledge without relying on a rehearsal buffer. Additionally, this paper introduces a robust benchmark to aid in the generalization of continual learning-based unsafe behavior recognition within the construction industry. Comparative experiments with other state-of-the-art approaches demonstrate our superior performance under challenging class-incremental settings.

## 2. Problem Statement

Emerging unsafe behavior recognition is defined as training machine learning models on a continuum of data from a sequence of recognition tasks. The sequence of tasks is denoted as $D=\left\{D_{1},\ldots ,D_{T}\right\}$, where the *t*-th task $D_{t}={\left\{\left(x_{i,t},y_{i,t}\right)\right\}}_{i=1}^{n_{t}}$ contains tuples of the input sample $x_{i,t}\in X$ and its corresponding label $y_{i,t}\in Y$. The recognition model $f_{\theta }:X\to Y$ is parameterized by θ such that it predicts the label $y=f_{\theta }\left(x\right)\in Y$ given an unseen test sample x from arbitrary tasks. Data from the previous tasks is not available when training future tasks.

This study relies on several commonly accepted assumptions. Firstly, it is assumed that task boundaries are distinct and task switching occurs abruptly during training. Secondly, the study adopts the challenging class-incremental learning setting, where task identity is unknown for each example during testing, closely mirroring real-world scenarios. Additionally, the availability of a pre-trained sequence model, such as a vision transformer trained on ImageNet, is presumed, aligning with prevalent practices in recent computer vision research. Notably, unlike many rehearsal-based approaches, our method does not require any form of rehearsal buffer.

## 3. Method

**Figure 1:**
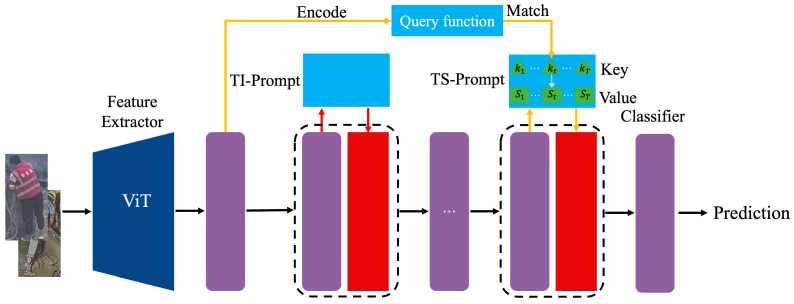
Overview of the proposed continual learning framework for emerging unsafe behavior recognition. The model mainly consists of a pre-trained visual backbone, dual prompt modules, and a classification head. Arrows indicate feature flow and knowledge retention or adaptation paths.

This method draws from prompt-based learning, which leverages learnable parameters—called prompts—to conditionally guide pre-trained models in performing downstream tasks. In our continual learning setting, we introduce two complementary prompt types: task-invariant prompts (TI-Prompts) and task-specific prompts (TS-Prompts). TI-Prompts are shared across all tasks and capture generalized knowledge beneficial to all future and past tasks. In contrast, TS-Prompts are specialized for each individual task and help isolate and retain task-specific information, thereby mitigating forgetting. During training, both prompts are inserted into selected layers of a frozen pre-trained transformer. The TI-Prompt remains constant across tasks, while the TS-Prompt changes according to the task identity. At inference time, we identify the most suitable TS-Prompt by matching the test sample to the closest task key, allowing the model to dynamically retrieve relevant knowledge. This dual-prompt architecture promotes continual learning by separating reusable and task-dependent features, offering both generalization and specialization. The proposed recognition framework is illustrated in Figure 1 Consider a pre-trained Vision Transformer (ViT) model, *f*, which comprises *N* sequential multi-head self-attention (MHSA) layers. We define the input embedding feature for the *i*-th MHSA layer as $h^{\left(i\right)}$, where *i* ranges from 1 to *N*.

### 3.1. Task Invariant Prompt

TI (Task Invariant)-Prompt, denoted as $I\in R^{L_{I}\times D}$, has a sequence length $L_{I}$ and an embedding dimension *D*, serving as a shared parameter across all tasks. When TI-Prompt is incorporated into the *i*-th MHSA layer, it modifies $h^{\left(i\right)}$ through a prompting function:

(1) $$h_{I}^{\left(i\right)}=f_{\mathrm{prompt}}\left(I,h^{\left(i\right)}\right),$$

where $f_{\mathrm{prompt}}$ determines the method of integrating the prompts into the hidden embeddings. Let $h\in R^{L\times D}$ represent the input to the MHSA layer, with $h_{Q}$, $h_{K}$, and $h_{V}$ being the input query, key, and value matrices, respectively. The prompt parameter I is divided into $I_{K}$ and $I_{V}\in R^{L_{I}/2\times D}$, which are then prepended to $h_{K}$ and $h_{V}$, leaving $h_{Q}$ unchanged:

(2) $$f_{\mathrm{prompt}}\left(I,h^{\left(i\right)}\right)=\mathrm{MHSA}\left(h_{Q}^{\left(i\right)},\left[I_{K}:h_{K}^{\left(i\right)}\right],\left[I_{V}:h_{V}^{\left(i\right)}\right]\right)$$

### 3.2. Task-Specific Prompt

While the TI-Prompt captures common knowledge across tasks, the task-specific prompt encodes unique information tailored for each task. TS(task-specific)-Prompt $S={\left\{s_{t}\right\}}_{t=1}^{T}$ consists of task-specific parameters, where each $s_{t}\in R^{L_{S}\times D}$ has a sequence length $L_{S}$ and an embedding dimension *D* matching that of the TI-Prompt. Here, *T* represents the total number of tasks. Unlike the shared TI-Prompt, every $s_{t}$ is paired with a task-specific key $k_{t}\in R^{D}$. This key is a learnable parameter designed to capture distinctive features of each task. For an input instance from the *t*-th task, we incorporate the TS-Prompt into the *j*-th MHSA layer using a similar prompting function:

(3) $$h_{s}^{\left(j\right)}=f_{\mathrm{prompt}}\left(s_{t},h^{\left(j\right)}\right).$$

Additionally, we refine the key $k_{t}$ to align with the input instance’s features through a matching loss $L_{\mathrm{match}}$, ensuring that $k_{t}$ becomes more representative of the *t*-th task compared to other keys. During testing, we employ a query function *q* on the test sample to identify the most suitable task key, subsequently selecting the corresponding TS-Prompt. We use the entire pre-trained model as the query function: q(x)=f(x)[0], with cosine similarity as γ. Therefore, the matching loss is expressed as follows:

(4) $$L_{\mathrm{match}\;}\left(x,k_{t}\right)=\gamma \left(q\left(x\right),k_{t}\right),\;x\in D_{t}.$$

To integrate the TS-Prompt with the embedding features similarly to the TI-Prompt, $s_{t}$ is divided into $s_{t,K}$ and $s_{t,V}$, each $\in R^{L_{S}/2\times D}$, and prepended to $h_{K}$ and $h_{V}$, while $h_{Q}$ remains unchanged:

(5) $$f_{\mathrm{prompt}}\left(s_{t},h^{\left(j\right)}\right)=\mathrm{MHSA}\left(h_{Q}^{\left(i\right)},\left[s_{t,K}:h_{K}^{\left(j\right)}\right],\left[s_{t,V}:h_{V}^{\left(j\right)}\right]\right).$$

### 3.3. Overall Objective

The architecture incorporating the prompts is represented by $f_{I,S_{t}}$. The input x from the *t*-th task is processed through $f_{I,S_{t}}$ and then passed to the classification head $f_{\phi }$, parameterized by ϕ, for prediction. Ultimately, both prompt types, task keys, and the initially random classification head are optimized in an end-to-end fashion:

(6) $$\min_{I,s_{t},k_{t},\phi }L_{\mathrm{CE}}\left(f_{\phi }\left(f_{I,s_{t}}\left(x\right)\right),y\right)+\lambda L_{\mathrm{match}\;}\left(x,k_{t}\right),\;x\in D_{t},$$

where $mathcal\;L_{\mathrm{CE}}$ is the cross-entropy loss and λ is a scalar balancing factor.

## 4. Split-UBR Dataset

**Figure 2:**
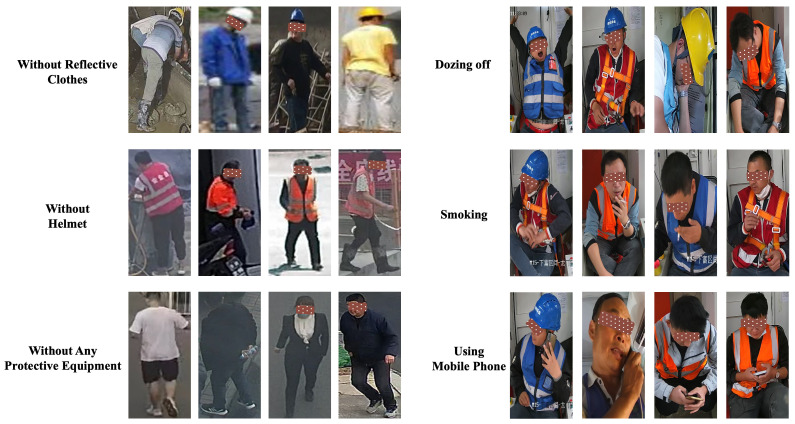
Representative samples from different unsafe behavior categories in the Split-UBR dataset. Each image is labeled according to site safety standards.

We introduce the Split Unsafe Behavior Recognition (Split-UBR) Dataset, designed to identify six classes of unsafe behavior on construction sites. The dataset is partitioned into training and test sets, consisting of 2400 and 600 images, respectively, all captured from real construction sites under varying conditions. The data were collected using handheld monocular cameras, site surveillance systems, and Unmanned Aerial Vehicles (UAVs), ensuring a diverse range of perspectives and conditions that reflect real-world challenges in construction environments. To support continual learning, the dataset is divided into three disjoint tasks, with two classes per task, allowing for incremental learning where models are exposed to new tasks sequentially. This structure encourages the development of algorithms that can adapt to new unsafe behavior categories without forgetting previously learned ones. Each task in the Split-UBR dataset reflects distinct types of unsafe behavior, such as the improper use of personal protective equipment or hazardous actions, allowing for comprehensive model evaluation across a variety of risk scenarios. By incorporating data from various sources and under different environmental conditions, Split-UBR simulates the complexities faced in real construction sites. This variability ensures that models trained on this dataset can generalize well to unseen data, addressing issues like occlusions, poor visibility, and diverse worker attire. The Split-UBR dataset is specifically designed to aid researchers in developing robust and adaptive algorithms for recognizing unsafe behaviors, enhancing construction site safety, and driving automation in construction processes. Several examples of unsafe behaviors are illustrated in Figure 2.

To provide a more comprehensive evaluation of the Split-UBR dataset, we first summarize the sample distribution across unsafe behavior categories. The dataset covers six distinct categories of unsafe behaviors, with samples evenly distributed across these classes. This balanced task structure eliminates class imbalance bias, thereby ensuring a fair assessment of continual learning algorithms. Representative annotated examples from each category are presented in Figure 2, which illustrate how the dataset captures visually diverse scenarios and contextual variability. To further assess the dataset’s variability, we analyzed the distribution of image acquisition conditions across three key dimensions: lighting conditions, camera perspectives, and environmental factors. Specifically, approximately 60% of the images were captured under natural daylight, 35% under artificial on-site lighting, and 5% under low-light or shadowed conditions. In terms of camera perspectives, around 80% of the samples were acquired from ground-level viewpoints, while the remaining 20% were captured via UAV-based aerial views. Additionally, the dataset incorporates scenes with dust, motion blur, and partial occlusions—factors that effectively simulate the complexity of real-world construction sites.

Split-UBR not only supports the development of AI-driven solutions for construction site safety but also serves as a benchmark for continual learning models. It addresses the challenge of handling dynamic environments where new categories of unsafe behaviors may emerge, necessitating adaptable and scalable recognition systems. Through its rich diversity of data sources, tasks, and annotations, the Split-UBR dataset offers a comprehensive foundation for advancing construction safety technologies in the Industry 5.0 era.

Ethics Statement. All procedures performed in studies involving human participants were in accordance with the ethical standards of the institutional and national research committee and with the 1964 Helsinki Declaration and its later amendments or comparable ethical standards. The study was approved by the Bioethics Committee of the Ocean University of China. Informed consent was obtained from all subjects involved in the study. Where applicable, explicit consent was also obtained for the publication of any potentially identifiable data or images in this open-access journal.

## 5. Experiment

### 5.1. Implementation Details and Evaluation Protocol

This section outlines the implementation details and comparative analysis results. The model is implemented using Pytorch 1.12.1 and trained on an NVIDIA GeForce RTX 3090 GPU, with each task undergoing 50 epochs of training. The batch size is set to 24, and the learning rate is 0.005. The backbone architecture adopted is the ViT-B/16 model. TI-Prompts are incorporated at the first and second MSA layers, whereas TS-Prompts are inserted at the third, fourth, and fifth MSA layers. To better handle ambiguous behaviors—instances that may not be clearly safe or unsafe—soft-label smoothing is applied during training to prevent overconfident predictions. In Equation (6), the coefficient λ controls the trade-off between the knowledge retention loss and the task adaptation loss. We empirically tuned λ on the validation split of the first incremental task within {0.1,0.3,0.5,0.7,1.0} and found that λ=0.5 consistently provided the best trade-off between stability and plasticity. Unless otherwise specified, λ is fixed to 0.5 for all experiments. Additionally, the prompt-based architecture facilitates more robust generalization by enabling the model to leverage both shared and task-specific contextual cues. For robust evaluation, experiments are repeated five times with different random seeds, reporting the average (Avg) and standard deviation (Std) of top-1 accuracy and forgetting [17].

### 5.2. Analysis of Model Architecture Design

**Figure 3:**
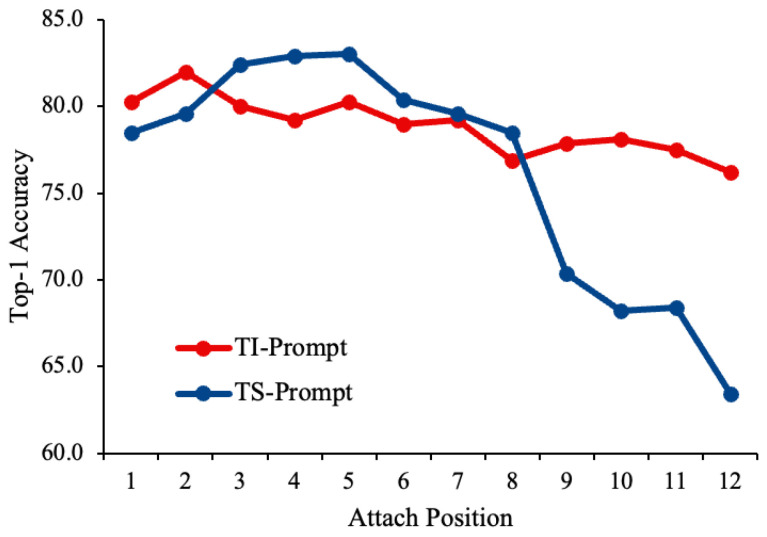
Effect of prompt insertion at different MSA layers on the Split-UBR dataset. The blue line denotes task-specific prompt performance, which peaks at the fifth MSA layer, while the orange line denotes task-invariant prompt performance, peaking at the second MSA layer.

A heuristic search strategy is employed on the Split-UBR dataset to determine the optimal insertion layer for both task-invariant and task-specific prompts within the Transformer backbone. As shown in Figure 3, the TS-Prompt achieves the highest performance when inserted at the fifth MSA layer, while the second MSA layer yields the best results for the TI-Prompt. This discrepancy indicates that TI-Prompts capture task-invariant knowledge more effectively at shallower layers, where more general representations are encoded, whereas TS-Prompts perform better at deeper layers, which contain task-dependent and discriminative information. Notably, both prompt types show performance degradation when inserted at the uppermost layers, suggesting that lower-level features allow more effective conditioning of the pre-trained model and contribute to better generalization across tasks. These results confirm that prompt placement plays a crucial role in balancing stability and plasticity in rehearsal-free continual learning.

### 5.3. Ablation Studies

The ablation study in Table 1 demonstrates the distinct contributions of TI-Prompt and TS-Prompt to continual learning performance. The baseline configuration, utilizing a frozen pre-trained backbone with a trainable classification head, achieves 65.7% Top-1 accuracy and 9.0% forgetting. This serves as the reference point for evaluating component efficacy. Introducing TI-Prompt alone significantly enhances performance, boosting accuracy by 16.2 percentage points to 81.9% while reducing forgetting by 2.9 percentage points to 6.1%. This improvement underscores TI-Prompt’s effectiveness in capturing task-invariant knowledge for cross-task generalization. However, the elevated forgetting standard deviation of 0.479 compared to the baseline 0.323 indicates the inconsistent mitigation of catastrophic forgetting across tasks, likely due to interference from shared representations. TS-Prompt alone further outperforms TI-Prompt, achieving 83.1% accuracy, an increase of 17.4 percentage points compared to the baseline, and 5.6% forgetting. Its remarkably low forgetting standard deviation of 0.159 demonstrates superior stability by isolating task-specific parameters, effectively preventing inter-task interference. Despite this advantage, TS-Prompt neglects the transferable knowledge benefits provided by task-invariant representations. The combined approach yields optimal results: 84.8% accuracy, an improvement of 1.7 percentage points compared to TS-Prompt alone, and 5.2% forgetting. This synergy decouples task-invariant and task-specific knowledge, effectively balancing stability, characterized by low forgetting, and plasticity, characterized by high accuracy. The marginal increase in standard deviations, with accuracy at 3.290 and forgetting at 0.401, suggests slight performance variability across tasks, but the combined approach robustly addresses the stability–plasticity dilemma inherent in continual learning scenarios.

### 5.4. Comparisons with the State of the Art

We compare our approach with representative baseline and state-of-the-art methods, including both rehearsal-based and rehearsal-free prompt-based continual learning approaches compatible with transformer architectures. Specifically, we include rehearsal-based methods such as Co2L [13] and DER++ [18], and prompt-based methods such as LwF [11], L2P [17], DualPrompt [19], and CODA-Prompt [20]. For accurate benchmarking, we also report the Lower-bound, representing naïve sequential training, and the Upper-bound, corresponding to standard supervised fine-tuning using data from all tasks. Table 2 summarizes the results on the Split-UBR dataset. Our proposed method achieves the highest average Top-1 accuracy of 86.9% with a remarkably low standard deviation of 2.502, indicating excellent stability across experimental runs. This performance not only exceeds rehearsal-based methods, Co2L with 82.2% and DER++ with 82.8%, but also surpasses recent rehearsal-free prompt methods. In particular, our method slightly outperforms CODA-Prompt with 86.8% and DualPrompt with 85.7%, demonstrating the effectiveness of our adaptive integration of task-invariant and task-specific prompts. In terms of forgetting, our approach achieves an exceptionally low average forgetting of 4.7, outperforming CODA-Prompt with 4.9 and DualPrompt with 5.3, as well as older baselines such as L2P with 7.9 and DER++ with 19.9. This highlights the superior knowledge retention capability of our method under rehearsal-free continual learning. Furthermore, the minimal standard deviation of 0.103 in forgetting indicates outstanding consistency and robustness. To further validate the robustness of the proposed approach, we conducted paired *t*-tests between our method and all baseline methods across five independent experimental runs. The results show that our method achieves statistically significant improvements (*p* < 0.05) in both Top-1 accuracy and forgetting rate compared with all baselines. This confirms that the observed performance gains are consistent and not due to random variations during training.

Overall, our method achieves superior performance without any buffered data, surpassing L2P by 5.2 percentage points in accuracy and maintaining the lowest forgetting across all methods. This success stems from the complementary interaction between task-invariant and task-specific prompts, which effectively disentangle generalizable and specialized knowledge to mitigate catastrophic forgetting. The performance gap between the Lower-bound with 53.1% and Upper-bound with 91.0% underscores the inherent difficulty of class-incremental learning, while our perform demonstrates significant progress toward the ideal upper limit.

### 5.5. Generalization Across Architectures

To verify the robustness and adaptability of the proposed dual-prompt learning framework, we further conducted experiments across five widely adopted backbone architectures: ViT-B/16 [21], Swin-Tiny [22], ConvNeXt-Tiny [23], ResNet-50 [24], and EfficientNet-B3 [25]. These architectures span both convolutional and transformer families, providing a comprehensive evaluation of architectural generalization. As shown in Table 3, our method achieves consistently high accuracy and low forgetting across all architectures. Transformer-based backbones, ViT-B/16 and Swin-Tiny, perform slightly better in Top-1 accuracy due to their superior global context modeling, while CNN-based architectures, ConvNeXt-Tiny, ResNet-50, and EfficientNet-B3, exhibit similarly low forgetting, demonstrating stable long-term retention. These results confirm that the proposed dual-prompt mechanism effectively enhances continual learning across different architectural designs, ensuring its adaptability for various deployment scenarios in construction safety monitoring.

### 5.6. Computational Efficiency and Deployment Considerations

To comprehensively assess the deployment feasibility of our proposed method in real-world construction safety monitoring systems, we conducted extensive performance benchmarks measuring both average inference time and GPU memory utilization on an NVIDIA RTX 3090 GPU with 24 GB of VRAM. The experimental setup involved processing high-definition video streams (1920 × 1080 resolution) under varying lighting conditions and scene complexities typically encountered in construction environments. Our model achieves an average processing speed of 30.2 frames per second (FPS), which exceeds the real-time performance threshold of 25 FPS required for most surveillance applications in the construction safety domain. This frame rate enables continuous monitoring without perceptible latency, allowing for the timely detection of potential safety hazards such as falls, improper equipment usage, or unauthorized personnel access to restricted areas.

Regarding resource consumption, the memory footprint during inference remains consistently under 5.4 GB, representing approximately 22.5% of the available VRAM on the test GPU. This efficient memory utilization makes our solution deployable on a wide range of modern GPUs and edge devices with high-performance compute capabilities, including mid-range graphics cards and specialized embedded AI accelerators. The integration of prompt-based mechanisms introduces minimal computational overhead, as these prompts are designed to be lightweight and are selectively applied only to specific layers within the network architecture. Our analysis indicates that the prompt-related operations account for less than 3% of the total computational budget, preserving the model’s efficiency while enhancing its adaptability to diverse safety monitoring scenarios.

## 6. Discussion

The study presented here represents a significant step forward in the use of artificial intelligence to enhance safety within the construction industry, with a particular focus on recognizing unsafe behaviors. This is a challenging domain due to the dynamic and unpredictable nature of construction sites. Traditional approaches to unsafe behavior recognition, which rely on predefined categories, are increasingly inadequate as new behaviors emerge [26,27]. Our proposed continual learning method, which integrates task-invariant and task-specific prompts, offers a promising solution. This model enables the recognition of both new and existing unsafe behaviors without the need for rehearsal buffers, a critical advancement in the context of data privacy concerns and scalability issues associated with traditional methods.

One of the key innovations of this research lies in its contribution to the field of continual learning, particularly in the domain of class-incremental learning. The integration of complementary prompts into pre-trained models not only mitigates catastrophic forgetting but also enhances task generalization. While prior approaches have focused on addressing catastrophic forgetting through various mechanisms—such as regularization, architecture modification, and rehearsal buffers—our approach leverages prompt-based learning, which provides a more flexible and scalable solution [28,29]. By separating task-invariant knowledge from task-specific knowledge, we facilitate the continual expansion of the model’s ability to recognize an ever-increasing set of unsafe behaviors. This innovation is especially relevant in real-world applications, where new types of unsafe behaviors may arise over time. The effectiveness of the proposed method, demonstrated by the superior performance on the Split-UBR dataset, highlights the robustness and adaptability of the approach. It shows that the model can maintain high accuracy even in class-incremental settings, where previous methods have struggled, particularly when faced with new categories.

### 6.1. Findings

In real-world construction sites, unsafe behaviors may not remain static but can evolve gradually or vary depending on contextual factors such as task type, environmental conditions, or team configurations [30]. Our model is designed to address this challenge by employing a dual-prompt strategy: the task-invariant prompt captures enduring safety-relevant patterns across tasks, while the task-specific prompt dynamically encodes knowledge unique to each task or stage of the learning process. This modular prompt mechanism enables the model to flexibly incorporate new behavior categories without overwriting previously learned knowledge. For instance, as new forms of unsafe behavior emerge due to shifts in workflow or equipment use, the TS-Prompt can rapidly adjust to these changes by learning new representations without requiring access to historical data. Meanwhile, the TI-Prompt ensures that core safety behaviors remain consistently recognized. Moreover, because prompts are lightweight and can be updated independently, the model can adapt to contextual behavior changes without retraining the entire backbone. This makes it especially suitable for deployment in environments with high variability, such as construction sites. Future research may further explore the dynamic weighting or gating mechanisms between TI and TS prompts to improve contextual sensitivity.

Another practical consideration is the model’s ability to handle ambiguous or borderline behaviors that do not clearly fall into either the “safe” or “unsafe” category. In real-world settings, such behaviors are common and may vary depending on environmental context, task urgency, or individual judgment [31]. Our architecture addresses this issue by leveraging both task-invariant and task-specific prompts. Task-invariant prompts capture general safety principles that remain consistent across scenarios, while task-specific prompts adapt to the contextual nuances of each task. This dual prompting mechanism enables the model to better interpret subtle cues, facilitating more nuanced classification of ambiguous behaviors. Additionally, the use of label smoothing during training helps the model remain less overconfident in uncertain cases, improving generalization and reducing the risk of misclassification in safety-critical applications.

The safety of workers on construction sites remains a significant concern globally, with a substantial portion of accidents attributable to unsafe behaviors. As construction environments are dynamic, the ability to adapt to newly emerging risks is crucial. Our method offers a framework for continuously learning from new data, which could significantly improve the real-time identification of unsafe behaviors. This, in turn, allows for more timely and targeted interventions, which are essential for preventing accidents before they occur. Moreover, the introduction of the Split-UBR dataset, with its realistic diversity of data sources and environmental conditions, provides a valuable resource for researchers in the field. This benchmark dataset not only facilitates the development of more sophisticated AI models but also sets a standard for evaluating the performance of continual learning algorithms in a construction context [32,33]. Future work could expand this dataset to include more diverse and complex behaviors, further improving the model’s capability to handle a wider range of real-world scenarios.

The effectiveness of the proposed method, demonstrated by the superior performance on the Split-UBR dataset, highlights the robustness and adaptability of the approach. It shows that the model can maintain high accuracy even in class-incremental settings, where previous methods have struggled, particularly when faced with new categories. Importantly, the model also exhibits promising scalability and deployment feasibility for real-time safety monitoring applications. Our experiments indicate that the model can operate at over 30 FPS on standard hardware, making it suitable for video-based safety analytics in live construction environments. Since prompt modules are injected only at the selected MSA layers and involve a small number of parameters, the resulting architecture maintains computational efficiency. This efficient design supports deployment in high-volume data scenarios, such as multiple surveillance streams across large-scale construction sites. Furthermore, the minimal inference overhead of prompts makes our method favorable for edge computing devices with limited resources, facilitating wide-scale and distributed deployment without sacrificing performance.

### 6.2. Limitations and Future Work

Despite the promising results, there are several limitations to this study that warrant future exploration. First, while our method effectively mitigates catastrophic forgetting in a rehearsal-free manner, it still faces challenges when dealing with highly imbalanced data or drastic domain shifts, which are common in real-world construction scenarios [34]. In such cases, the fixed representations learned by the pre-trained backbone may offer limited generalization, and task-specific prompts may be biased toward dominant categories, leading to reduced adaptability. To address these issues, future research could explore adaptive weighting mechanisms to dynamically balance class-level losses, domain-adaptive prompting strategies conditioned on environment-specific features, or hybrid continual learning frameworks that combine our rehearsal-free paradigm with lightweight generative replay or meta-learning modules. These directions could further enhance the model’s robustness under evolving or heterogeneous data distributions. Second, the reliance on pre-trained vision models, while effective in leveraging existing knowledge, could be limiting in terms of task-specific fine-tuning. Investigating more flexible training paradigms, such as few-shot learning or meta-learning [35,36], could strengthen the model’s capacity to adapt rapidly to novel unsafe behaviors with limited supervision. Lastly, the integration of multimodal data [37], including audio, sensor inputs, and behavioral signals from wearable devices, could provide a richer contextual foundation for unsafe behavior recognition. Future iterations of the model could thus explore multimodal fusion to build more comprehensive and context-aware safety monitoring systems capable of identifying subtle and complex risk patterns in dynamic construction environments.

The application of AI technologies to monitor worker behavior, while beneficial for improving safety outcomes, raises important ethical concerns, particularly related to privacy and surveillance. Construction workers should not be subjected to intrusive or opaque monitoring systems. To address these concerns, any deployment of the proposed system must comply with ethical guidelines and legal standards. This includes ensuring informed consent, where workers are clearly notified about how monitoring systems operate, what data is collected, and for what purposes. Data collected must be anonymized and securely stored, with access restricted to authorized personnel to safeguard privacy. The system’s operations and decision-making processes should be transparent and auditable to foster accountability and trust. Moreover, monitoring should be strictly limited to safety enhancement purposes and not be used for productivity tracking or disciplinary actions. Importantly, workers should be actively involved in the deployment process to ensure that their rights, perspectives, and concerns are fully respected. By implementing these safeguards, AI-based monitoring systems can support improved workplace safety while maintaining individual autonomy and dignity. Future research should also consider the broader socio-technical implications and incorporate participatory studies involving key stakeholders to further validate ethical deployment practices.

## 7. Conclusions

This paper presents a novel rehearsal-free continual learning framework for emerging unsafe behavior recognition in the construction industry, addressing a critical challenge in maintaining safety awareness under dynamic and evolving work conditions. The proposed method enhances a pre-trained vision transformer by integrating complementary task-invariant (TI) and task-specific (TS) prompts, effectively balancing the stability–plasticity trade-off. This design enables the model to retain previously learned knowledge while efficiently adapting to new unsafe behavior categories without relying on rehearsal data. In addition to the methodological innovation, we introduce Split-UBR, a new benchmark dataset specifically designed for continual unsafe behavior recognition in construction environments. Split-UBR provides a realistic task structure with incremental class emergence, facilitating rigorous evaluation of continual learning algorithms in safety-critical domains. Extensive experimental results demonstrate that our approach achieves state-of-the-art performance compared to both rehearsal-based and rehearsal-free methods, exhibiting superior accuracy, reduced forgetting, and strong robustness under data stream variations. The dual-prompt strategy not only improves representation learning but also offers interpretability regarding how task-invariant and task-specific knowledge evolve through model layers.

Overall, this study provides both theoretical and practical advances toward human-centered and resilient construction safety monitoring, aligning with the vision of Industry 5.0 for intelligent, adaptive, and ethically responsible workplace automation. Future research will focus on extending the framework to multimodal sensor fusion and cross-site generalization, further broadening its applicability in real-world construction safety management.

## Figures and Tables

**Table 1:** Ablation study on TI-Prompt and TS-Prompt.

TI-Prompt	TS-Prompt	Top-1 Accuracy		Forgetting	
		Avg	Std	Avg	Std
		65.7	2.057	9.0	0.323
✓		81.9	2.237	6.1	0.479
	✓	83.1	3.063	5.6	0.159
✓	✓	84.8	3.290	5.2	0.401

**Table 2:** Results on class-incremental learning.

Method	Top-1 Accuracy		Forgetting	
	Avg	Std	Avg	Std
Lower-bound	53.1	6.371	36.0	1.994
Upper-bound	91.0	0.520	-	-
Co2L [13]	82.2	2.673	23.4	2.212
DER++ [18]	82.8	5.074	19.9	1.691
LwF [11]	66.2	13.998	25.2	3.373
L2P [17]	81.7	7.486	7.9	2.833
DualPrompt [19]	85.7	3.866	5.3	0.744
CODA-Prompt [20]	86.8	2.326	4.9	0.203
Ours	86.9	2.502	4.7	0.103

**Table 3:** Generalization analysis of the proposed method across different backbone architectures. Results are reported in terms of Top-1 accuracy and forgetting on the Split-UBR dataset.

Backbone	Top-1 Accuracy (%)	Forgetting (%)
ViT-B/16 [21]	86.9	4.7
Swin-Tiny [22]	87.3	4.5
ConvNeXt-Tiny [23]	86.8	4.6
ResNet-50 [24]	86.0	4.9
EfficientNet-B3 [25]	86.4	4.8

## Data Availability

The datasets used or analyzed during the current study are available from the corresponding author on reasonable request.
